# Supplementary material for: Design and performance of an oversized-sample 35 GHz EPR resonator with an elevated Q value
Source: Magn Reson (Gott). 2024 Sep 20;5(2):143–52. doi: 10.5194/mr-5-143-2024 (PMC12178130; doi:10.5194/mr-5-143-2024)
Supplement: The supplement related to this article is available online at: https://doi.org/10.5194/mr-5-143-2024-supplement. [file mr-5-143-2024-supplement.pdf]

Supplement of Magn. Reson., 5, 143–152, 2024
https://doi.org/10.5194/mr-5-143-2024-supplement
© Author(s) 2024. CC BY 4.0 License.

Supplement of

Design and performance of an oversized-sample 35 GHz EPR resonator with an elevated Q value

Jörg Wolfgang Anselm Fischer et al.

Correspondence to: Daniel Klose (daniel.klose@phys.chem.ethz.ch)

The copyright of individual parts of the supplement might differ from the article licence.

1 Microwave characteristics from S_{11} curves: Half-power points and VSWR

The resonance frequency ν_{dip} is the frequency of the dip center in the S_{11} curves. The bandwidth of the resonator is the frequency difference between the half-power points after baseline correction to remove waveguide losses (see Fig. S1). The half-power points dB_{hp} (in dB) of the S_{11} curves, which are shifted from the -3 dB points when the resonator is not critically coupled, are given by (Eaton et al., 2010, p. 86)

$$dB_{\text{hp}} = 10 \cdot log\left(1 - \frac{1 - 10^{dB_{\text{dip}}/10}}{2}\right) , \tag{S1}$$

where dB_{dip} is the magnitude of the S_{11} curve at the dip center relative to the baseline offset due to waveguide losses. The loaded Q value, Q_L, is calculated as the ratio between the resonance frequency ν_{dip} and the bandwidth (BW) measured between the half-power points $\nu_{\text{hp,i}}$ (frequency values of dB_{hp} given above):

$$Q_L = \frac{\nu_{\text{dip}}}{\nu_{\text{hp,2}} - \nu_{\text{hp,1}}} . \tag{S2}$$

The Voltage Standing Wave Ratio (VSWR) of the resonator is calculated by

$$\text{VSWR} = \frac{1 + 10^{S11dB/20}}{1 - 10^{S11dB/20}} . \tag{S3}$$

The beta coefficient β is defined as VSWR = $1/\beta$ for undercoupled resonators ($\beta < 1$), and as VSWR $= \beta$ for overcoupled resonators ($\beta > 1$). The coefficient β further allows to calculate the unloaded Q value, Q_0, from Q_L, independent from critical coupling, as

$$Q_0 = Q_L \cdot (1 + \beta) . \tag{S4}$$

A schematic drawing illustrating the determination of these parameters from the S_{11} curve can be found in Fig. S1. All parameters were obtained for the empty resonator and for the resonator with an empty 3 mm clear fused quartz tube inserted at room temperature.

2 Filling factor calculation

The filling factor is the ratio of the magnetic field component that induces the EPR signal over the overall energy stored in the resonator. (Misra, 2011) It is calculated by

$$\eta = \frac{\int_{\text{sample}} B_{1,\text{rot}}^2 \text{d}V}{\int_{\text{cavity}} B_1^2 \text{d}V} , \tag{S5}$$

where $B_{1,rot}$ is the magnetic field in the rotating frame and B_1 is the magnetic field strength. $B_{1,\text{rot}}$ is calculated as half of the magnetic field components orthogonal to B_0, since for linearly polarized microwave irradiation only half of the B_1 amplitude in the laboratory frame leads to excitation of EPR transitions. (Misra, 2011)

3 Supplementary Tables and Figures

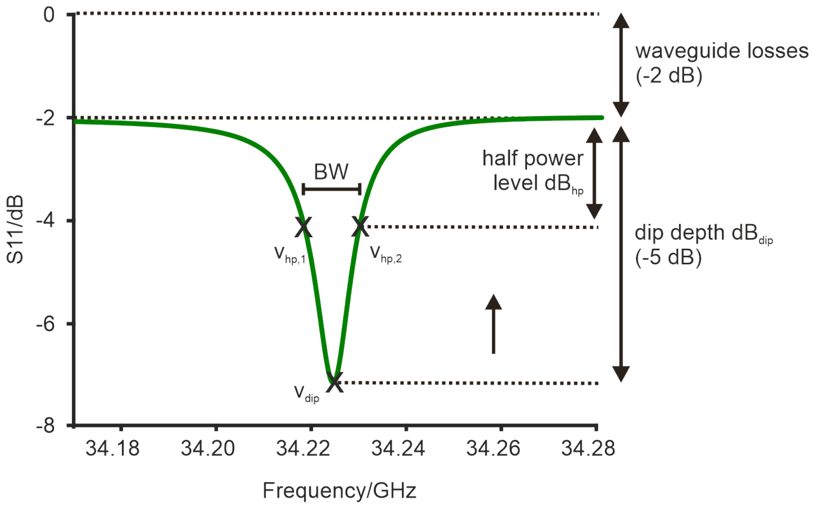

Figure S1. Schematic S_{11} microwave reflection curve and annotated parameters used to assess microwave characteristics.

Table S1. Measurement parameters of the conducted Q-band CW EPR experiments. The parameters were chosen for optimal signal-to-noise, while avoiding saturation of the receiver/detector of the spectrometer. In all cases a modulation frequency of 100 kHz was used. The effective microwave power, P_{eff}, was measured before the probehead by a power meter. The nominal power, P_{nom}, was shown by the spectrometer software.

Measurement	Width / mT	Modulation amp. / mT	Conv. time / ms	Time const. / ms	P_{eff} / μW	P_{nom} / μW
DPPH	5	0.02	81.92	20.48	0.32	0.3
10 ppm N@C60	2	0.002	160	40.96	0.23	2.0
Ti(III) r.t.	60	0.1	161.84	20.48	0.57	5.0
Ti(III) 30 K	100	0.1	81.92	20.48	0.23	2.0

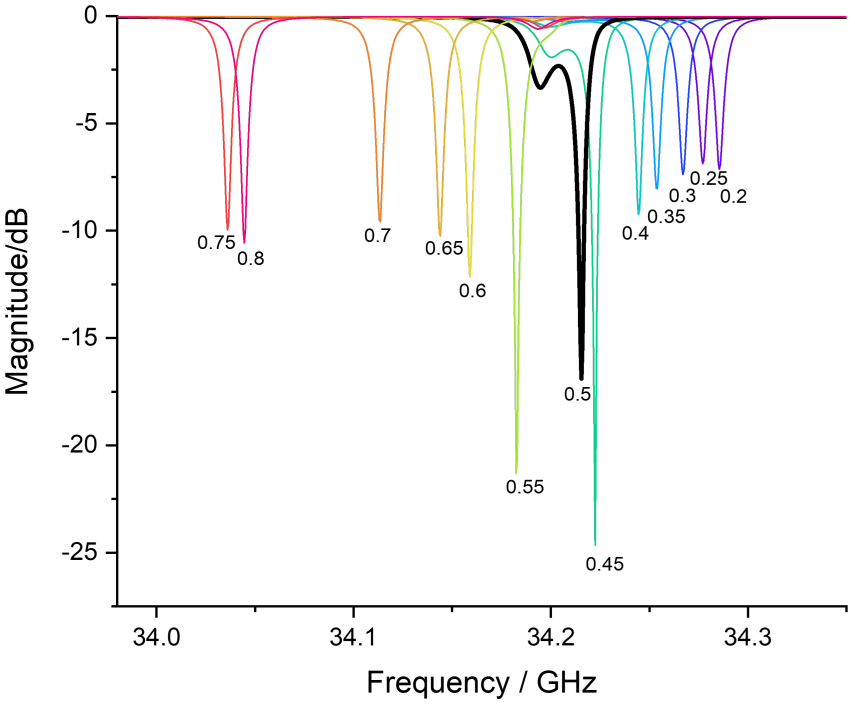

Figure S2. Simulated S_{11} curves of the resonator with slot widths between 0.2 and 0.8 mm, while the slot centers were held 1 mm apart. The resonator dip has a maximal depth for slot widths of 0.45 - 0.55 mm. The central, selected slot width (0.5 mm) is highlighted in black. The individual slot widths are annotated in millimeters.

Table S2. Dip parameters from simulated S_{11} curves of cavities with varying height and diameter containing a 3 mm quartz tube filled with a high-dielectric sample (6 mm, $\epsilon = 2.5$). Dimensions of entry 2 (9.0 x 11.5 mm) were selected for manufacturing due to the minimum S_{11} value.

Height / mm	Diameter / mm	Frequency / GHz	Bandwidth / MHz	$S11_{dip}$ / dB
8.5	11.7	34.26	8.3	-8.5
9	11.5	34.22	8.8	-26.0
9.5	11.3	34.24	9.1	-8.6
10	11.2	34.18	8.6	-8.5
10.5	11.1	34.15	8.5	-8.3
11	11	34.16	8.6	-8.3
13	10.8	34.08	7.9	-8.3
15	10.7	34.02	7.2	-9.4
15	11.6	34.22	7.8	-9.6

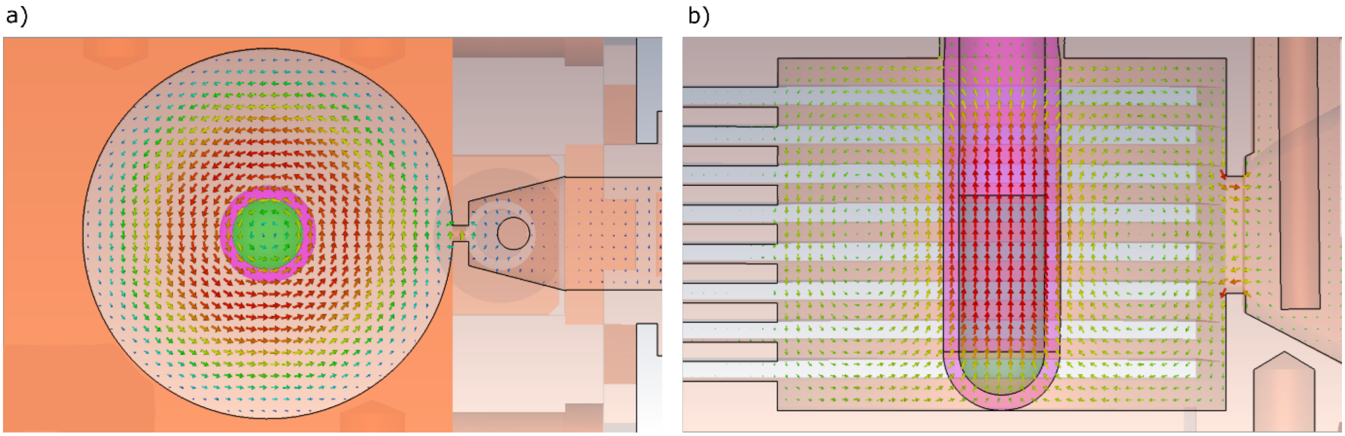

Figure S3. Electromagnetic field simulations of the resonator with a tube filled with a medium with a dielectric constant of $\epsilon = 2.5$. The resulting electric vector field (a) and the corresponding magnetic vector field (b) are shown as colored arrows (red to blue for large to zero fields).

Table S3. Comparison of filling factors η for two resonators, the TE_{011} resonator and the previously published TE_{102} resonator (Tschaggelar et al., 2009), for samples with a range of dielectric constants ϵ. Sample filling height in the quartz tube is 5 mm.

Resonator mode	$\epsilon = 1.0$	$\epsilon = 1.5$	$\epsilon = 2.0$	$\epsilon = 2.4$	$\epsilon = 2.7$	Reference
TE_{011}	0.057	0.061	0.066	0.070	0.074	This work
TE_{102}	0.023	0.024	0.029	0.032	0.034	Tschaggelar et al., 2009

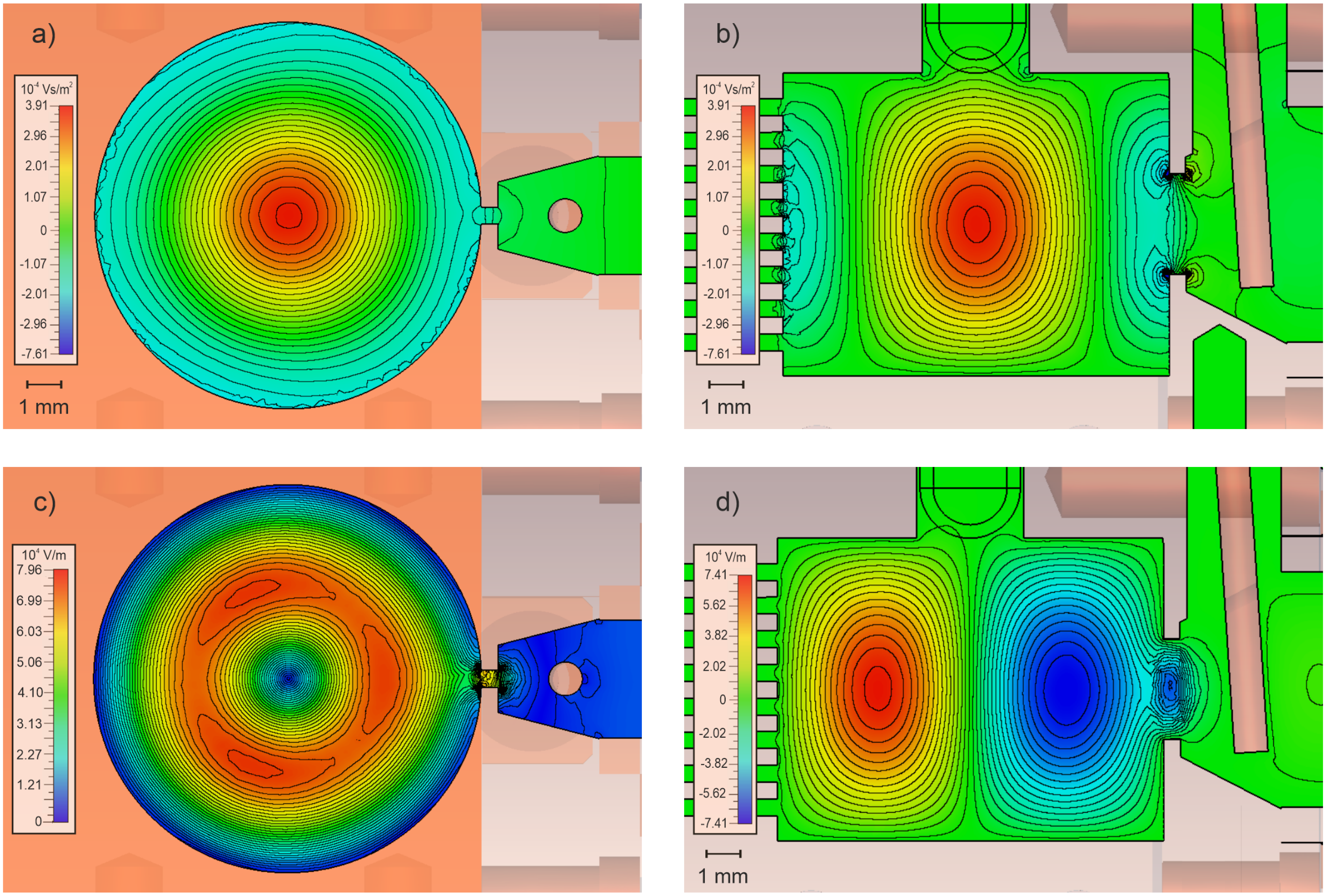

Figure S4. B and E field simulations of the resonator without EPR tube, isolines are spaced by 6 % steps of the maximum field strength, all cross sections run through the center of the cavity. (a) Strength of the x component of the B_1 field in a horizontal cross section, (b) Strength of the x component of the B_1 field in a vertical cross section, (c) Strength of the tangential component of the E_1 field in a horizontal cross section, (d) Strength of the z component of the E_1 field in a vertical cross section.

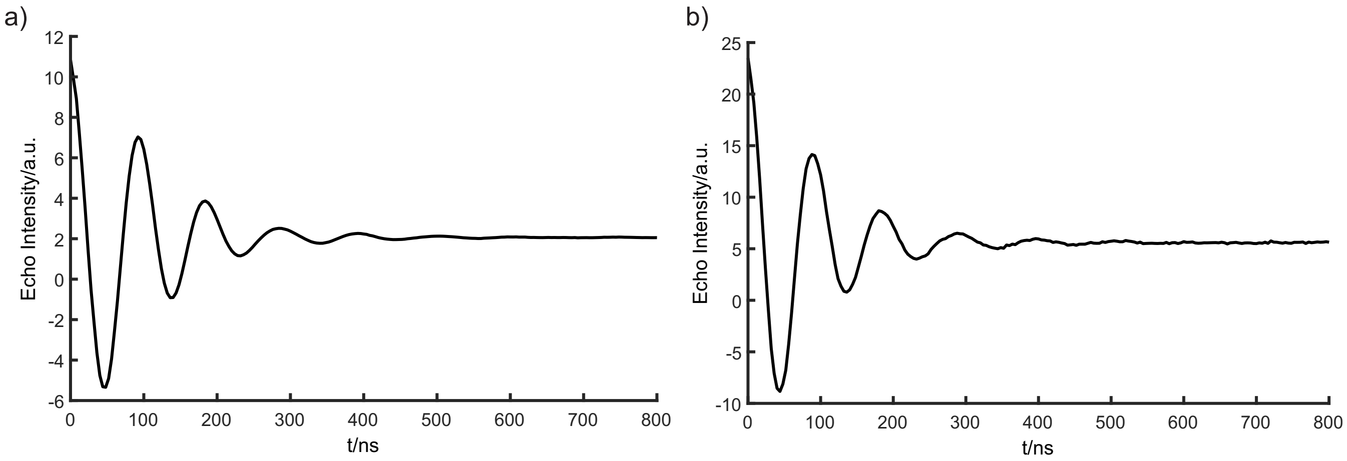

Figure S5. EPR nutation experiments on γ-irradiated Herasil at 33.934 GHz at a B_0 corresponding to the spectral maximum with a pulse sequence t_P - 3 µs - $\pi/2$ (24 ns) - 1 µs - π (48 ns) - 1 µs - echo at room temperature (a) and at 50 K (b). The Q-factor of 2500 ± 100 was measured beforehand with the sample centered in the cavity. For the room temperature measurement in (a) a shot repetition time of 1 ms and a measured input power of 0.74 W was used, resulting in a π pulse length of 46 ns and a calculated conversion factor of 0.48 mT/$\sqrt{\mathrm{W}}$. For the measurement at 50 K in (b) the shot repetition time was 200 ms and the input power 0.60 W, resulting in a π pulse length of 44 ns and a calculated conversion factor of 0.52 mT/$\sqrt{\mathrm{W}}$. Nutation experiments on Ti(III) catalysts in frozen toluene solutions showed similar conversion factors (0.39 - 0.53 mT/$\sqrt{\mathrm{W}}$).

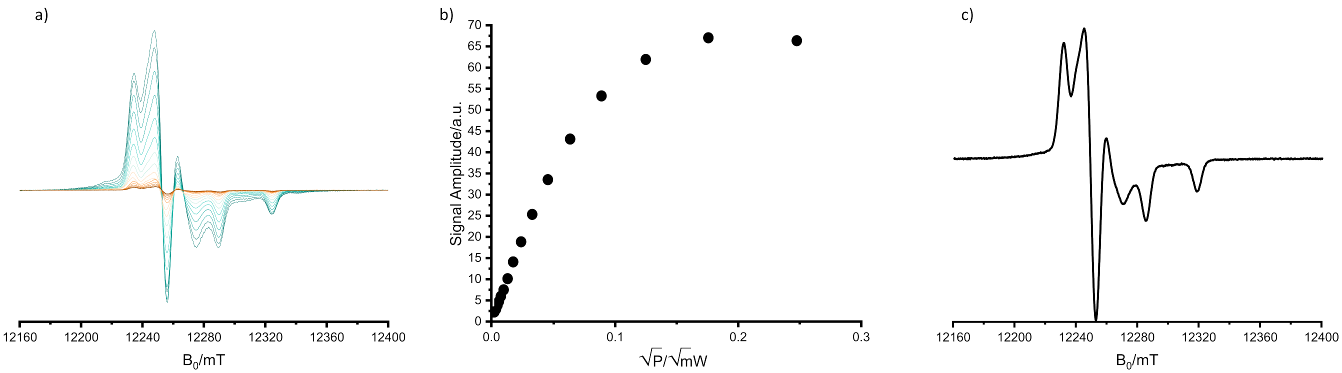

Figure S6. CW EPR power saturation at 100 K of 100 μM TEMPOL radical in toluene (20 μl) with increasing microwave power from brown to green (a) and the corresponding peak-to-peak signal amplitudes as a function of square root of effective microwave power delivered to the probehead (b). The measurement parameters are: Sweep width: 25 mT, modulation frequency: 100 kHz, modulation amplitude: 0.1 mT, conversion time: 80 ms, time constant: 40 ms. Spectrum used for determining the signal-to-noise ratio of 471 in 4 scans under non-saturating conditions (c), using the peak-to-peak intensity as the signal and two standard deviations over baseline points as the noise intensity, with measurement parameters: Sweep width: 25 mT, modulation frequency: 100 kHz, modulation amplitude: 0.1 mT, conversion time: 80 ms, time constant: 40 ms, nominal microwave power: 10.02 μW, corresponding to 1.1 μW effective microwave power (see Tab. S1).
